# Editorial: Cardiac Vibration Signals: Old Techniques, New Tricks, and Applications

**DOI:** 10.3389/fphys.2022.931362

**Published:** 2022-06-17

**Authors:** Kouhyar Tavakolian, Omer T. Inan, Jin-Oh Hahn, Marco Di Rienzo

**Affiliations:** ^1^ Biomedical Engineering Program, University of North Dakota, Grand Forks, ND, United States; ^2^ College of Engineering, Georgia Institute of Technology, Atlanta, GA, United States; ^3^ Robert E. Fischell Institute for Biomedical Devices, University of Maryland, College Park, MD, United States; ^4^ Fondazione Don Carlo Gnocchi Onlus (IRCCS), Milan, Italy

**Keywords:** seismocardiography (SCG), phonocardiography (PCG), photoplethysmogram (PPG), ballistocardiogram (BCG), cardiac vibration signals

## Introduction

Every heartbeat sets the body into a series of vibrations that can be recorded using transducers placed on the surface of the body, such as wearables, or the surfaces that are in contact with the body, such as chairs, beds, or weight scales. These vibrations have been studied for more than a century and the corresponding signals have been given different names depending on the method of recording and the sensor placement. Currently, Phonocardiography (PCG), Ballistocardiography (BCG), and Seismocardiography (SCG) are the signals most commonly considered in this area, although other signals or signal combinations may also be considered.

Since the start of the new millennium, there has been a particular burst in the research on these signals, in part triggered by the recent advances in the sensor miniaturization and signal processing techniques. In some instances, these signals have even been integrated into medical devices. However, a lot of work is still needed to improve recording and analysis of these signals, fully understand the genesis of their waveforms, and verify their applicability in the clinical practice. The goal of this article Research Topic is to bring together new research ideas on each of these challenges.

## Novel Recording Methods

Implantable systems could provide a possibility to obtain high-quality, real-world, and longitudinal data, that do not require the involvement of the patient to correctly and regularly acquire cardiac vibration signals. The feasibility of acquiring useful electrocardiographic (ECG) and accelerometry data from an innovative implant located in the gastric fundus was investigated by Areiza-Laverde et al. In the first phase of the study, data were simultaneously acquired in the gastric fundus and by traditional surface sensors on two pigs. In the second phase, the feasibility of deriving useful hemodynamic markers from these gastric signals was explored. Results showed a high correlation between parameters acquired from the gastric and thoracic sites, as well as pre-clinical evidence on the feasibility of chronic cardiovascular monitoring from an implantable cardiac device located at the gastric fundus.

In another effort, a new recording method is proposed by Andreozzi et al. using a particular piezoelectric-based force sensor to record both the lower and higher frequency vibration signal from the chest. A beat-to-beat morphological comparison was done with respect to Photoplethysmography (PPG) and SCG signals and close similarity with these signals was established.

## The Genesis of Waves

The genesis of waves in BCG has been a topic of research from long ago. A comprehensive review of the early works in this area may be found in (Starr and Noordergraaf, 1967). However, the original models were based on classical simplified approaches and lacked the currently available computational power needed to fully explore the complexity of the problem. In this Research Topic of papers, two articles addressed this issue using modern modelling approaches.

Rabineau et al. proposed an exhaustive and accurate computational BCG model including a closed-loop 0D-1D multiscale representation of the human blood circulation. The 0D elements included the cardiac chambers, cardiac valves, arterioles, capillaries, venules, and veins, while the 1D elements included 55 systemic and 57 pulmonary arteries. In this model, the contributions of the cardiac chambers and the pulmonary circulation are non-negligible, especially on the lateral and transversal velocity BCG signals.

A physiology-based mathematical model coupled with evolutionary algorithms was presented by Marazzi et al. to obtain personalized estimates of cardiovascular parameters. A combination of ECG and BCG signals was used to capture amplitudes and timings of the most prominent peak and valley in the BCG curve, also known as the J peak and K valley and estimate blood pressure and parameters pertaining to ventricular function. This paper follows the previous effort by the same group in BCG modeling using the traditional ultra-low-frequency BCG (Guidoboni et al., 2019).

## Clinical Relevance

The clinical relevance of cardiac vibration signals has always been a topic of attention. In this Research Topic, four papers focused on the clinical or physiological relevance of these signals.

Extraction of cardiac time intervals (CTI), and in particular systolic time intervals, has been an established area of research in cardiac vibration signals, particularly using SCG and PCG fiducial points (Dehkordi et al., 2019). Nevertheless, in cardiac patients the extraction of these timings may be difficult because the heart dysfunction can modify the SCG waveform so to mask the typical fiducial points needed for their estimation. This aspect has been addressed in a study by Zeybek et al., including ninety patients with myocardial infarction, heart failure, or transplanted hearts. Results indicate that not every cardiac patient has a SCG waveform suitable for the CTI estimation. Thus, it is suggested that before starting a SCG-based CTI monitoring, a preliminary check by a simultaneous SCG-echocardiographic measure is advisable to verify the applicability of the methodology to a specific patient.

Detection of coronary artery disease from SCG signals goes back to early 1990s (Salerno and Zanetti, 1991). Dehkordi et al. further investigated this issue by considering SCG and Gyrocardiography (GCG) signals at rest and analyzing data by a one-dimensional Convolutional Neural Network. The predicted CAD risk was validated against related results from angiography. Classifiers based on the dorsoventral and longitudinal components of SCG showed better performance relative to the other candidates. The sensitivity range for CAD detection was 92%–94% for the SCG-based classifiers and 73%–87% for the GCG-based classifiers. Moreover, these findings showed that the performance of the proposed 3-axial SCG/GCG solution based on recordings obtained during rest was comparable to or better than stress ECG.

Making recorded cardiac vibration signals available online could significantly benefit the field. An open-access database for SCG and GCG signals is proposed in by Yang et al. The archive comprises SCG and GCG recordings collected and processed at multiple sites worldwide, including Columbia University Medical Center and Stevens Institute of Technology in the United States, as well as Southeast University, Nanjing Medical University, and the first affiliated hospital of Nanjing Medical University in China. It includes ECG, SCG, and GCG recordings collected from 100 patients with various conditions of valvular heart diseases such as aortic and mitral stenosis.
